# Versorgung von Menschen mit Seltenen Erkrankungen: Empfehlungen für eine gelungene intersektorale Zusammenarbeit

**DOI:** 10.1007/s00103-023-03719-y

**Published:** 2023-06-13

**Authors:** Laura Inhestern, Maja Brandt, Ramona Otto, David Zybarth, Martin Härter, Corinna Bergelt

**Affiliations:** 1 Institut und Poliklinik für Medizinische Psychologie, Universitätsklinikum Hamburg-Eppendorf, Martinistr. 52, 20246 Hamburg, Deutschland; 2 Institut für Medizinische Psychologie, Universitätsmedizin Greifswald, Greifswald, Deutschland

**Keywords:** Schnittstellen, Versorgungsforschung, Mixed-methods-Design, Versorgungsstrukturen, Diagnose, Interfaces, Health services research, Mixed-methods design, Healthcare structures, Diagnosis

## Abstract

**Hintergrund:**

Seltene Erkrankungen (SE) sind häufig durch komplexe Beschwerdebilder charakterisiert und erfordern in der Regel im Diagnose- und Versorgungsverlauf die Zusammenarbeit von spezialisierten Zentren und Primärversorgenden. Reibungslose Schnittstellen mit geringem Informationsverlust und Kooperation stellen daher eine essenzielle Grundlage in der Versorgung dar. Das Projekt „Evaluation von Schnittstellenmanagementkonzepten bei Seltenen Erkrankungen“ (ESE-Best) verfolgte mittels verschiedener Erhebungsinstrumente das Ziel, Empfehlungen für die Gestaltung und Umsetzung von Schnittstellen in der Versorgung von Menschen mit Seltenen Erkrankungen zu entwickeln.

**Methoden:**

Es wurden mittels quantitativer und qualitativer Befragungen die Perspektiven der Zentren für Seltene Erkrankungen (ZSE), der Primärversorgung und der Betroffenen (Patient:innen, Eltern) erfragt sowie 2 Expert:innen-Workshops durchgeführt.

**Ergebnisse:**

Es wurden insgesamt 28 Empfehlungen in den folgenden 5 Bereichen formuliert: 1) Vernetzung zwischen Primärversorgung und Zentren für Seltene Erkrankungen (ZSE), 2) Schnittstellen innerhalb der ZSE, 3) Bekanntheit von Seltenen Erkrankungen, ZSE-Strukturen und Zuständigkeiten, 4) Schnittstellen zwischen ZSE und Patient:innen sowie 5) weiterführende Empfehlungen.

**Diskussion:**

Die Empfehlungen sollen zukünftig zu einem funktionierenden Schnittstellenmanagement bei der Versorgung von Menschen mit Seltenen Erkrankungen beitragen. Da die Erfahrungswerte von Primärversorgenden, ZSE und Betroffenen in die Entwicklung der Empfehlungen eingeflossen sind, können die externe Validität und damit die Umsetzbarkeit im Alltag angenommen werden. Es ist zu bedenken, dass zeitliche und personelle Ressourcen sowie organisationale Strukturen die Schnittstellenarbeit im Einzelfall beeinflussen können. Die Empfehlungen können an örtliche Gegebenheiten adaptiert werden.

## Abstract (en)

**Background:**

Rare diseases often present complex symptoms and usually require intersectoral collaboration during diagnostic and therapeutic processes involving inpatient and outpatient care. Hence, smooth interfaces with little loss of information and cooperation are essential to provide appropriate care. Our study, the project ESE-Best, aims at developing recommendations for the design and implementation of intersectoral care for patients with rare diseases using various survey instruments.

**Methods:**

Using quantitative and qualitative methods, multiple perspectives (primary physicians, expert centers of rare diseases, patients, parents) were assessed. Additionally, two expert workshops were conducted.

**Results:**

Based on findings from our data, we formulated 28 recommendations in the following areas: (1) networking between primary physicians and expert centers, (2) intersections within the expert centers, (3) awareness of rare diseases, structures of expert centers and responsibilities, (4) collaboration between expert centers and patients/caregivers, and (5) further recommendations.

**Conclusion:**

Our recommendations provide a basis for a working management of intersectoral care in rare diseases. As the recommendations are based on broad data including multiple perspectives, external validity and feasibility can be assumed. Still, time and human resources as well as organizational structures in single centers or practices and regional structures need to be taken into account as they may impact intersectoral care.

## Einleitung

Eine Erkrankung wird als selten definiert, wenn weniger als 5 von 10.000 Menschen betroffen sind (Europäische Kommission, 2008). Aktuell sind etwa 6000 bis 9000 Seltene Erkrankungen (SE) bekannt [1, 2], wobei jährlich etwa 150 bis 250 weitere Erkrankungen hinzukommen [1]. Es wird angenommen, dass bei etwa 3–8 % der Bevölkerung mindestens eine Seltene Erkrankung diagnostiziert wurde. Für Deutschland kann entsprechend eine Anzahl von 2,5–5 Mio. Menschen mit einer Seltenen Erkrankung geschätzt werden [2, 3].

Seltene Erkrankungen sind häufig durch komplexe Beschwerdebilder charakterisiert und können schwerwiegende körperliche und kognitive Funktionseinschränkungen sowie eine reduzierte Lebenserwartung für die Betroffenen bedeuten. Bei einem Großteil der Erkrankungen ist ein Symptombeginn im Kindesalter zu beobachten [1]. Derzeit geht man bei etwa 72 % der Seltenen Erkrankungen von einer genetischen Ursache aus [4], wobei diese Anzahl sich aufgrund dynamischer Entwicklungen und verbesserter genetischer Diagnosemethodik stetig verändert.

Die Klärung der Diagnose und der Krankheitsursache mittels genetischer Untersuchungen oder anderer diagnostischer Möglichkeiten stellt in der Regel die Grundlage für eine bedarfs- und erkrankungsorientierte Versorgung der Betroffenen dar [5, 6]. Trotz der verbesserten diagnostischen Möglichkeiten wird der Diagnoseweg von Betroffenen häufig als eine lange Odyssee bis zur richtigen Diagnose beschrieben. Die Suche nach der Diagnose und adäquater Versorgung kann dabei zu hoher Belastung führen [7] und ist in der Regel mit vielen Kontakten im Gesundheitssystem und praktischen wie auch emotionalen Herausforderungen verbunden [8].

Auch wenn eine Diagnose gestellt wurde, ist die weitere Versorgung aufgrund der begrenzt verfügbaren regionalen Spezialversorgung sowohl für die Betroffenen als auch für die ärztlichen Versorgenden eine große Herausforderung [9]. Neben begrenzten regionalen Spezialangeboten fehlen häufig kurative Therapieoptionen, sodass ausschließlich eine symptomorientierte Behandlung erfolgt, wofür der Einbezug unterschiedlicher Spezialist:innen und Fachgebiete dauerhaft notwendig ist [10]. Zudem stellen regelmäßige Kontrolltermine oder notwendige unterstützende Maßnahmen eine hohe Anforderung an das Erkrankungs- bzw. Gesundheitsmanagement der Betroffenen dar. Das Krankheitsmanagement umfasst beispielsweise die Koordination von Terminen mit unterschiedlichen Spezialist:innen oder die Aufklärung und Informationsweitergabe an andere Fachkräfte, die an der Versorgung beteiligt sind (Ärzt:innen, aber auch z. B. weiterführend therapeutisch Tätige). Von ärztlicher Seite wird jedoch berichtet, dass insbesondere die Kommunikation zwischen den verschiedenen Fachbereichen und Leistungssektoren häufig unzureichend und die Versorgung dadurch beeinträchtigt ist [11].

Basierend auf den Empfehlungen des Europäischen Rats zu Strategien in Bezug auf die Anforderungen in der Versorgung von Menschen mit Seltenen Erkrankungen wurden auch für Deutschland im Nationalen Aktionsplan für Menschen mit Seltenen Erkrankungen (NAMSE) Strategien und Empfehlungen erarbeitet, um die Bedarfe (z. B. in der Diagnosestellung und Therapie) zu adressieren [12]. Durch die Einrichtung spezialisierter Zentren für Seltene Erkrankungen (ZSE) verändert sich die Versorgungslandschaft für die Betroffenen und es wird Kompetenz generiert, strukturiert und aggregiert. Ein zentraler Aspekt in der Ausgestaltung der Zentrenstruktur sind die sogenannten Referenzzentren (Typ-A-Zentren) als koordinierende Zentren und Anlaufstelle für Menschen mit Verdacht auf eine Seltene Erkrankung, die bisher noch keine Diagnose erhalten haben. Den Referenzzentren zugehörig sind Fachzentren bzw. -ambulanzen (Typ-B-Zentren) zur Versorgung einzelner Erkrankungsbereiche. Auch niedergelassene Fachärzt:innen können z. B. durch Behandlungsschwerpunkte als enge Partner:innen der Zentren fungieren (Typ-C-Zentren). Die ZSE werden beispielsweise im SE-Atlas dargestellt (www.se-atlas.de), in dem neben den Versorgungsangeboten der Zentren im Allgemeinen auch Hinweise zu den Angeboten für einzelne seltene Erkrankungsgruppen aufgeführt werden.

Trotz dieser positiven Entwicklungen hin zu einer verbesserten spezialisierten Versorgung ist die Versorgung von Patient:innen mit Seltenen Erkrankungen in besonderem Maße durch Schnittstellenprobleme gekennzeichnet. Im NAMSE wird darauf hingewiesen, dass insbesondere für die Diagnosestellung die Zusammenarbeit zwischen den Zentren und Primärversorgenden zentral ist [12]. Schnittstellendefizite in der ärztlichen Versorgung können darüber hinaus die Versorgungsqualität erheblich beeinträchtigen [13–15]. Schnittstellenmanagement umfasst die gezielte Kommunikation und abgestimmte Zusammenarbeit aller an der Versorgung Beteiligten [16]. Ein gelungenes Schnittstellenmanagement zwischen Institutionen und Versorgenden wird in der Versorgungsforschung [17] und Gesundheitspolitik seit Langem als Herausforderung für eine qualitätsgesicherte medizinische Versorgung thematisiert. Verbesserungspotenzial wird u. a. in der Etablierung eines strukturierten Entlassmanagements sowie in der Kommunikation und der interdisziplinären Zusammenarbeit gesehen [15, 18–20]. Das Ärztliche Zentrum für Qualität im Gesundheitswesen (ÄZQ) hat dazu dezidierte Empfehlungen herausgegeben [21]. Aus den dargestellten Besonderheiten bei der Diagnostik und Versorgung Seltener Erkrankungen wird deutlich, dass das Thema Schnittstellen für alle Beteiligten (Patient:innen bzw. Eltern von minderjährigen Patient:innen, Haus- und Fachärzt:innen, ZSE) zentral ist.

In dem vom Bundesministerium für Gesundheit (BMG) geförderten Forschungsprojekt ESE-Best („Evaluation von Schnittstellenmanagementkonzepten bei Seltenen Erkrankungen“) wurden auf Basis einer multiperspektivischen Mixed-Methods-Studie Empfehlungen für das Schnittstellenmanagement in der ärztlichen Versorgung von Menschen mit Seltenen Erkrankungen entwickelt.

## Methoden

**Figure 1:**
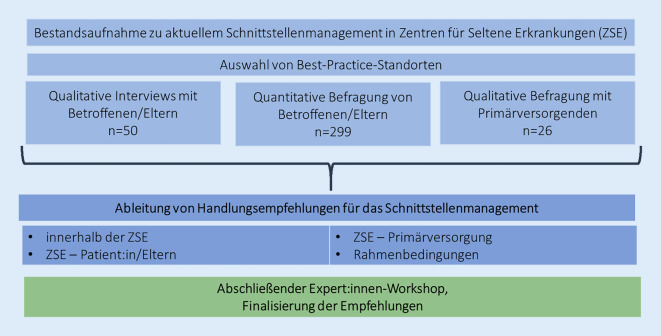

Die Empfehlungen werden auf Basis von Erkenntnissen aus insgesamt 3 Projektphasen erarbeitet. Dabei wurden ein multimethodaler Ansatz mit qualitativen und quantitativen Methoden sowie ein konsequenter Einbezug der Perspektiven der an der Versorgung Beteiligten (ZSE, Primärversorgung, Patient:innen, Eltern) umgesetzt (Abb. 1).

In der ersten Studienphase erfolgte eine Bestandsaufnahme des Schnittstellenmanagements in der Versorgung von Menschen mit Seltenen Erkrankungen. Es wurde auf Basis von Literaturrecherchen, eines Expert:innen-Workshops, Vor-Ort-Visitationen und Expert:innen-Interviews ein Schema zur Erfassung unterschiedlicher Bereiche des Schnittstellenmanagements entwickelt. Dieses Schema wurde in 2 Versionen (ZSE, Primärversorgung) ausgearbeitet und umfasste die folgenden 7 Aspekte: Bekanntheit des ZSE, Kommunikation mit Patient:innen, Aufnahme in das ZSE, Kommunikation mit Primärversorgenden, Abläufe innerhalb des ZSE, Weiterbehandlung/Entlassung aus dem ZSE, Zusammenarbeit mit Patient:innenorganisationen. Das Schema wurde in einer telefonischen Befragung von Vertreter:innen von ZSE und Primärversorgenden eingesetzt. Zum Projektbeginn wurden 32 ZSE über den SE-Atlas identifiziert und zu einer Teilnahme eingeladen. Neben der koordinierenden Stelle der ZSE (A-Zentrum/Referenzzentrum) sollten 3–4 Vertreter:innen einer Spezialsprechstunde (B-Zentrum/Spezialambulanz) befragt werden. Darüber hinaus wurden Primärversorgende, die mit den ZSE kooperieren, befragt.

Auf Basis der Aussagen der teilnehmenden A‑Zentren/Referenzzentren der ZSE wurden den Zentren Punkte für die o. g. 7 Aspekte vergeben. Einzelne Items wurden dabei im Rahmen des Expert:innen-Workshops, der Interviews oder der Vor-Ort-Visitationen als besonders relevant eingestuft und doppelt gewichtet. Der maximal erreichbare Gesamtpunktwert lag bei 87 Punkten (A-Zentrum) bzw. 89 Punkten (B-Zentrum). In einem zweiten Schritt wurden die Aussagen und Punkte der A‑Zentren mit denen der teilnehmenden B‑Zentren der ZSE abgeglichen. Der Median der erreichten Punktwerte der A‑Zentren lag bei 60. Es wurden 6 Zentren, die über dem Median lagen und damit im Gesamtwert sowie in einzelnen Bereichen durch ein funktionierendes Schnittstellenmanagement gekennzeichnet waren, als „Best-Practice-Standorte“ identifiziert und in einer zweiten Studienphase zu einer weiteren Studienteilnahme eingeladen.

In der zweiten Studienphase wurde die Patient:innenperspektive zum Schnittstellenmanagement in den ausgewählten Standorten erfragt. 3 der 6 ausgewählten Best-Practice-Standorte folgten der Einladung zur Teilnahme an der zweiten Studienphase. An diesen Standorten (Göttingen, Hamburg, Heidelberg) wurden über das Referenzzentrum sowie über Spezialsprechstunden/-ambulanzen, die von den ZSE bestimmt wurden, Patient:innen bzw. die Eltern von minderjährigen Patient:innen für eine schriftliche Befragung gewonnen. Darüber hinaus wurden Patient:innen und Eltern von einzelnen Patient:innenorganisationen zur Studienteilnahme eingeladen. Neben Fragen zur Befindlichkeit (erfasst mit der Hospital Anxiety and Depression Scale – HADS; [22]) und zur Lebensqualität (erfasst mit dem WHOQOL BREF der Weltgesundheitsorganisation [23]) wurden dabei Fragen zu den Unterstützungsbedürfnissen (in Anlehnung an den Supportive Care Needs Survey [24], bei Eltern erkrankter Kinder ergänzend die Parental Needs Scale [25]) gestellt sowie schnittstellenrelevante Fragen zur Versorgung am ZSE (selbst entwickelte Items basierend auf der ersten Studienphase, z. B. Kontaktaufnahme und Erreichbarkeit der Ansprechperson). Weitere Fragen bezogen sich auf die Zusammenarbeit der Behandelnden (in Anlehnung an [26]) und auf die Zufriedenheit mit der Versorgung (erfasst mit dem Fragebogen zur Zufriedenheit in der ambulanten Versorgung Schwerpunkt Patient:innenbeteiligung – ZAPA [27] und dem Fragebogen zur Patient:innenzufriedenheit ZUF‑8 [28]). Bei Eltern wurden außerdem die Pflegebelastung und die pflegebezogene Lebensqualität [29, 30] sowie die Lebensqualität der Kinder [31] miterhoben.

Ergänzend wurden in dieser Studienphase halbstrukturierte qualitative Interviews mit einer Teilstichprobe der Betroffenen sowie mit Primärversorgenden geführt, um die Erfahrungen mit der Versorgung und speziell dem Schnittstellenmanagement vertiefend zu erfassen. Zentrale Themenbereiche im Interviewleitfaden für die Betroffenenbefragung waren 1) Diagnoseweg, 2) Zugangsweg zum ZSE, 3) Erfahrung mit der Diagnostik bzw. Behandlung am ZSE, 4) Wahrnehmung der Zusammenarbeit zwischen ZSE und Primärversorgenden (Haus- und Fachärzt:innen), 5) Verbesserungswünsche in der Versorgung, 6) Unterstützungsbedarfe, 7) Einfluss der Coronapandemie auf die Versorgung/Erkrankung. Die Betroffeneninterviews dauerten zwischen 16 min und 84 min (durchschnittlich 43 min).

Folgende Themenbereiche wurden bei den Interviews mit den Primärversorgenden vertieft: 1) Bekanntheit der ZSE, 2) Gründe für und Ablauf der Anmeldung von Patient:innen am ZSE, 3) Kommunikation mit dem ZSE während und nach der Behandlung durch das Zentrum, 4) langfristige Zusammenarbeit mit dem ZSE, 5) Verbesserungswünsche und -vorschläge für die Zusammenarbeit.

Die quantitativen Ergebnisse der Patient:innenbefragung wurden deskriptiv und mithilfe von Gruppenvergleichen ausgewertet. Für die Ableitung der Empfehlungen waren insbesondere die schnittstellenrelevanten Fragen zu Erfahrungen mit der Versorgung, Erfahrungen zur Zusammenarbeit der Behandelnden und zur Zufriedenheit mit der Versorgung zentral. Die qualitativen Interviews wurden mithilfe der qualitativen Inhaltsanalyse nach Mayring mit der Software MAXQDA (VERBI Software. Consult. Sozialforschung GmbH, Berlin, Deutschland) ausgewertet.

Im letzten Schritt wurden die Ergebnisse der ersten beiden Studienphasen zu ersten Entwürfen der Empfehlungen aufbereitet und im Rahmen eines abschließenden Expert:innen-Workshops diskutiert und überarbeitet. Nach einer schriftlichen Konsentierungsrunde und einer erneuten Feedbackschleife wurden die finalen Empfehlungen formuliert.

## Ergebnisse

### Datengrundlage der Empfehlungen

#### Bestandsaufnahme.

Im Rahmen der Bestandsaufnahme wurden 4 Expert:innen-Interviews, 4 Vor-Ort-Visitationen in ZSE und 1 Expert:innen-Workshop durchgeführt. Am Expert:innen-Workshop nahmen 4 Vertreter:innen aus 4 ZSE, 6 Vertreter:innen von Patient:innenorganisationen sowie 2 Vertreter:innen des Bundesministeriums für Gesundheit (BMG) teil. Basierend auf den Ergebnissen wurde ein Schema zur Erfassung relevanter Bereiche des Schnittstellenmanagements gemeinsam mit Vertreter:innen der Selbsthilfe, eines ZSE, des NAMSE sowie des BMG konsentiert. In der Befragung von insgesamt 70 Vertreter:innen aus 24 ZSE (A- und B‑Zentren) und 42 Primärversorgenden wurde das Schema dann angewandt.

#### Befragung an Best-Practice-Standorten.

Auf Basis der Ergebnisse der Bestandsaufnahme wurden die Best-Practice-Standorte ausgewählt. In die Befragung wurden an 3 Standorten sowie über Patient:innenorganisationen insgesamt *n* = 299 Betroffene (*n* = 176 Patient:innen; *n* = 123 Eltern minderjähriger Patient:innen) einbezogen. 65 % der Befragten sind weiblich, das durchschnittliche Alter liegt bei 45 Jahren. Fast alle Betroffenen haben die deutsche Staatsangehörigkeit. Ein Großteil der Befragten ist verheiratet und kann der sozialen Mittel- und Oberschicht zugeordnet werden. 76 % der Patient:innen bzw. der Kinder haben eine gesicherte Diagnose einer SE erhalten.

Ergänzend stellten die qualitativen Daten von *n* = 50 Betroffenen (*n* = 38 Patient:innen; *n* = 12 Eltern) und *n* = 26 Primärversorgenden in den Auswertungen eine zentrale Grundlage für die Empfehlungen dar.

#### Synthese der Ergebnisse.

Auf Basis der Ergebnisse aus der Bestandsaufnahme und der Betroffenen- und Primärversorgendenbefragung wurden durch das Projektteam die relevanten Aspekte für das Schnittstellenmanagement geprüft und ergänzt. Darüber hinaus wurde auf Basis der Beurteilungen durch die Betroffenen und Primärversorgenden abgeleitet, welche Schnittstellenlösungen funktionieren, welche Faktoren dies beeinflussen und welche Lösungs- und Verbesserungsmöglichkeiten bereits umgesetzt werden bzw. umgesetzt werden sollten. Darauf basierend wurde ein erster Entwurf möglicher Empfehlungen durch das Projektteam erstellt. Der Entwurf wurde auf dem abschließenden Expert:innen-Workshop (digital) vorgestellt, an dem 4 Vertreter:innen der ZSE, 4 Vertreter:innen von Patient:innenorganisationen (inkl. ACHSE e. V.) und 2 Vertreter:innen des NAMSE teilnahmen. Eine Vertreterin des BMG nahm als nicht stimmberechtigter Gast am Workshop teil. Auf Basis von Rückmeldungen im Workshop wurden die Empfehlungen überarbeitet und schließlich in einer schriftlichen Feedback- und Konsensrunde finalisiert.

### Abgeleitete Empfehlungen für ein gelungenes Schnittstellenmanagement

Die im Projekt entwickelten und abschließend abgeleiteten und konsentierten Empfehlungen für ein gelungenes Schnittstellenmanagement beziehen sich auf verschiedene in der Versorgung auftretende Schnittstellen. Insgesamt wurden 8 Empfehlungen zur Vernetzung zwischen Primärversorgung und ZSE und 5 Empfehlungen zu Schnittstellen innerhalb der ZSE sowie 6 Empfehlungen zu Schnittstellen zwischen ZSE und Patient:innen abgeleitet. Darüber hinaus wurden 4 Empfehlungen zur Steigerung der Bekanntheit von Seltenen Erkrankungen, ZSE-Strukturen und Zuständigkeiten und 5 weiterführende Empfehlungen für das Schnittstellenmanagement abgeleitet (Tab. 1).

**Table 1:** 

**Vernetzung zwischen Primärversorgung und Zentren für Seltene Erkrankungen (ZSE)**	
*Empfehlungen für ZSE*	
Empfehlung 1:	ZSE sollten direkte Kontaktmöglichkeiten (z. B. ärztliche Durchwahlnummern, gesonderte Sprechzeiten, E‑Mail-Kontakt mit zeitnaher Rückmeldung) und Anmeldemöglichkeiten von Patient:innen (Kurzanmeldung, Terminvereinbarung durch Praxen) für Primärversorgende vorhalten. Diese sollten regelmäßig auf Aktualität überprüft werden
Empfehlung 2:	ZSE sollten Primärversorgende über Vorgaben zu notwendigen Unterlagen und Untersuchungen sowie zur Überweisung von Patient:innen informieren und (bei Bedarf und/oder Notwendigkeit sowie mit Einverständnis der Patient:innen) Unterlagen direkt bei Primärversorgenden anfordern
Empfehlung 3:	ZSE sollten zuweisende Primärversorgende und Patient:innen über erfolgte Behandlungsschritte sowie die weiteren Behandlungspfade zeitnah informieren. Berichte (Arztbriefe, Befunde) sollten kompakt formuliert zeitnah an Zuweisende und Patient:innen übermittelt werden und Hinweise enthalten, worauf bei der Versorgung von Patient:innen mit entsprechenden Erkrankungen zu achten ist
Empfehlung 4:	ZSE sollten für die Versorgung gemeinsamer Patient:innen die Möglichkeit zum direkten Austausch für Primärversorgende anbieten, z. B. in Form von (Online‑)Sprechstunden, Fallkonferenzen und/oder Qualitätszirkeln
*Empfehlungen für Primärversorgende*	
Empfehlung 1:	Hausärzt:innen bzw. Kinderärzt:innen sollten, sofern dies im Rahmen der Versorgung indiziert ist, die Rolle als Behandlungskoordinierende für die Versorgung von Patient:innen mit Seltenen Erkrankungen außerhalb des ZSE übernehmen und sich mit dem ZSE und anderen beteiligten Versorgenden direkt austauschen
Empfehlung 2:	Primärversorgende sollten direkte Kontaktmöglichkeiten (z. B. ärztliche Durchwahlnummern, gesonderte Sprechzeiten) für ZSE sowie andere beteiligte Versorgende vorhalten bzw. eine zeitnahe Rückmeldung sicherstellen
Empfehlung 3:	Primärversorgende sollten für die Versorgung gemeinsamer Patient:innen die Möglichkeit zum direkten Austausch mit den ZSE nutzen, z. B. in Form von (Online‑)Sprechstunden, Fallkonferenzen und/oder Qualitätszirkeln, sofern dies im Rahmen der Versorgung indiziert ist
Empfehlung 4:	Primärversorgende sollten für die Versorgung von Menschen mit Seltenen Erkrankungen sensibilisiert werden und hierzu an akkreditierten (Online‑)Fortbildungen von ZSE teilnehmen, z. B. zu Themen wie ZSE-Strukturen, Indikatoren für Überweisungen sowie ggf. Erkrankungsgruppen oder „red flags“^a^
**Schnittstellen innerhalb der ZSE**	
Empfehlung 1:	ZSE sollten eine zentrale Stelle zur Koordination und zur Weiterleitung der Patient:innen an die B‑Zentren bereithalten. Es sollte ein engmaschiger Austausch zwischen A‑Zentrum und B‑Zentren bestehen sowie durch das A‑Zentrum eine direkte Anbindung von Patient:innen an die B‑Zentren erfolgen (inkl. zentraler Terminvergabe durch die koordinierte Stelle)
Empfehlung 2:	Der Datentransfer innerhalb der Zentrumsstruktur sowie zwischen dem ZSE und anderen behandelnden Einrichtungen innerhalb der Organisation, in der das ZSE angesiedelt ist, sollte digital erfolgen
Empfehlung 3:	ZSE sollten ein Transitionskonzept für den Übergang von der Kinder- in die Erwachsenenmedizin vorhalten und umsetzen
Empfehlung 4:	ZSE sollten für die verschiedenen Prozesse (z. B. Aufnahme, Weiterleitung, Entlassung) standardisierte Konzepte bereithalten (z. B. in Form von Standard Operation Procedures – SOPs) sowie Checklisten für die Abläufe innerhalb des Zentrums entwickeln und nutzen (z. B. Sichtung eingehender Unterlagen, Datentransfer, Registermeldung)
Empfehlung 5:	ZSE sollten sich bei entsprechender Spezialisierung an der Teilnahme und dem Ausbau von Deutschen Referenznetzwerken (DRN) und Europäischen Referenznetzwerken (ERN) beteiligen
**Steigerung der Bekanntheit von Seltenen Erkrankungen, ZSE-Strukturen und Zuständigkeiten**	
Empfehlung 1:	ZSE sollten Informationsmaterial bereithalten (z. B. übersichtliche Homepage, Flyer, kompakte Beschreibungen, Hinweise zu Veranstaltungen am Tag der Seltenen Erkrankungen) und dieses, sofern über entsprechende Verteiler möglich (z. B. der Fachgesellschaften), an (regionale) Primärversorgende/Fachärzt:innen und relevante Fachverbände weitergeben. Die Materialien sollten enthalten: a) Informationen zur Struktur des ZSE (wie Anmeldungsprozesse, Kriterien zur Aufnahme von Patient:innen und Beschreibungen zu Versorgungspfaden im ZSE) b) Informationen zu den im ZSE versorgten Seltenen Erkrankungen bzw. Erkrankungsgruppen (ggf. mit Hinweisen zu „red flags“) oder Hinweise auf weitere existierende Informationen (z. B. Plattformen wie se-atlas, Orphanet und ACHSE e. V., Leitlinien)
Empfehlung 2:	A‑Zentren der ZSE sollten die o. g. Informationen transparent, barrierefrei und leicht auffindbar auf ihrer Website präsentieren. Es sollten mindestens die folgenden Kerninformationen vorgehalten werden: a) Kontaktdaten von Ansprechpartner:innen, b) Ablauf der Anmeldung, c) Schwerpunkte und zugehörige B‑Zentren, d) Umgang mit unklaren Diagnosen
Empfehlung 3:	ZSE sollten regelmäßige, akkreditierte (Online‑)Fortbildungen für alle an der medizinischen und psychosozialen Versorgung beteiligten Professionen anbieten, z. B. zu Themen wie ZSE-Strukturen, Erkrankungsgruppen oder „red flags“
Empfehlung 4:	Die ZSE sollten eine Vernetzung untereinander fördern, z. B. in Form von regelmäßigen Veranstaltungen für die Lots:innen^b^
**Schnittstellen zwischen ZSE und Patient:innen**	
Empfehlung 1:	ZSE sollten sich zum Ziel setzen, kurze Wartezeiten für Patient:innen zu ermöglichen. Insbesondere Anliegen von Patient:innen mit einer hohen Dringlichkeit sollten priorisiert und zeitnah versorgt werden
Empfehlung 2:	ZSE sollten ausreichende und zuverlässige Kontaktmöglichkeiten und Ansprechpartner:innen bereithalten, um die regelmäßige (telefonische) Erreichbarkeit (auch in Urlaubs- und Krankheitsfällen) für Patient:innen zu gewährleisten
Empfehlung 3:	Patient:innen sollten Informationen zur Verfügung gestellt werden, in denen Zuständigkeiten und Ansprechpartner:innen für die medizinische Versorgung, psychologische Begleitung und sozialrechtliche Beratung bzgl. ihrer jeweiligen Seltenen Erkrankung aufgeführt werden (z. B. Hinweise auf Plattformen wie se-atlas, Orphanet, ACHSE e. V. und vorhandene regionale Angebote in Form von Flyern)
Empfehlung 4:	ZSE sollten Patient:innen auf erkrankungsspezifische Patient:innenorganisationen und auf die ACHSE e. V., auch bei unklaren Seltenen Erkrankungen, hinweisen (z. B. durch die Erwähnung in Arztbriefen). B‑Zentren sollten hierzu aktuelle Listen mit Patient:innenorganisationen zu den versorgten Erkrankungsgruppen führen. A‑Zentren sollten eine Zusammenarbeit mit der ACHSE e. V. pflegen
Empfehlung 5:	ZSE sollten Patient:innen eine zeitnahe und verbindliche Rückmeldung zu ihren Anfragen geben (z. B. erfolgte Anmeldung, Bearbeitungsstand, nächste Schritte)
Empfehlung 6:	ZSE sollten ihre Unterlagen (auch) in patient:innengerechter Sprache zur Verfügung stellen, ggf. unter Nutzung von Angeboten wie „patientenbriefe.de“
**Weiterführende Empfehlungen für das Schnittstellenmanagement**	
Empfehlung 1:	Es sollten Strukturen geschaffen werden, die die Patient:innen bei der Organisation und Koordination der Versorgung bedarfsorientiert und individuell unterstützen (z. B. Lots:innen, Case- und Care-Manager:innen). Die genaue Ausgestaltung und Implementierung solcher Strukturen sollten wissenschaftlich geprüft werden
Empfehlung 2:	Bestehende Informationsstrukturen (z. B. se-atlas) sollten genutzt bzw. ausgebaut werden, um sowohl für Patient:innen als auch für Versorgende das Auffinden spezialisierter Ansprechpartner:innen zu erleichtern und somit wohnortnahe Versorgung durch stärkere Vernetzung zu ermöglichen
Empfehlung 3:	Eine zentrale und zeitnahe elektronische Erfassung erfolgter Behandlungen zur Informationsweitergabe an (Mit‑)Behandelnde und Patient:innen sollte ermöglicht werden
Empfehlung 4:	Bestehende zentrale und niedrigschwellige Informationsportale (wie z. B. se-atlas) zu ZSE-Standorten und Strukturen für Primärversorgende und Betroffene sollten verstetigt und kontinuierlich gepflegt werden
Empfehlung 5:	Deutsche Referenznetzwerke (DRN) sollten für alle Seltenen Erkrankungen bzw. Erkrankungsgruppen sowie für unklare Seltene Erkrankungen geschaffen werden. Bestehende DRN und Europäische Referenznetzwerke (ERN) sollten weiter ausgebaut werden und ZSE bei entsprechender Spezialisierung zur Teilnahme verpflichtet werden

^**a**^ Als „red flag“ werden Hinweise, z. B. Symptomkonstellationen, verstanden, die auf eine SE hindeuten

^b^ Lots:innen sind i. d. R. in den ZSE tätig und i. d. R. eine der ersten Ansprechpersonen bei (Verdachts‑)Diagnosen bzw. unklaren Diagnosen

## Diskussion

Vor dem Hintergrund der Herausforderungen in der Diagnostik und Versorgung von Menschen mit Seltenen Erkrankungen, die im Rahmen der Zentrenstruktur wie auch durch wohnortnahe Primärversorgung stattfindet und in der Regel neben der medizinischen Versorgung weiterführende Therapieangebote beinhaltet, sind reibungslose und gut etablierte Abläufe notwendig. Eine funktionierende Schnittstellengestaltung kann dabei essenziell dazu beitragen, a) Diagnosewege zu beschleunigen (u. a. Verdachtsdiagnosen, Befundübermittlung) und b) bei erfolgter Diagnosestellung die Versorgung möglichst so zu gestalten, dass Zusammenarbeit und Informationsweitergabe problemlos und ohne übermäßige Verantwortung aufseiten der Patient:innen möglich ist. Die im NAMSE vorgeschlagene und inzwischen umgesetzte Zentrenstruktur stellt dabei eine wichtige Grundlage dar [12].

Im Rahmen des ESE-Best-Projekts wurden Empfehlungen für die Gestaltung von Schnittstellen abgeleitet. Dies erfolgte auf Basis der aktuellen Versorgungssituation und der praktisch umgesetzten Versorgung in den ZSE und der Primärversorgung. Im Statusbericht zur Umsetzung des NAMSE aus 2019 wird darauf hingewiesen, dass die Ergebnisse des Projekts ESE-Best in den NAMSE-Prozess zur Ausgestaltung der Zusammenarbeit zwischen ZSE und Primärversorgenden, insbesondere hinsichtlich der Diagnosestellung, berücksichtigt werden, um Strategien zu entwickeln [32]. Die abgeleiteten Empfehlungen ergänzen die Ergebnisse des Projekts TRANSLATE NAMSE zur Verbesserung des Diagnosewegs durch strukturierte diagnostische Pfade [33, 34].

Durch einen geregelten und direkten Austausch zwischen ZSE und Primärversorgenden zu gemeinsamen Patient:innen können Maßnahmen abgestimmt, die Behandlungsplanung abgesprochen und ggf. doppelte Untersuchungen vermieden werden. Integrierte Versorgungsansätze erfordern in der Regel Ressourcen auf beiden Seiten, können aber möglicherweise im Verlauf der Behandlung zu Zeit- und Kostenersparnissen führen (z. B. durch Verringerung/Verhinderung von Komplikationen oder von zusätzlichen Ärzt:innenbesuchen aufgrund von Verunsicherung; [35]). Inwieweit auch die elektronische Patientenakte (ePA) übergreifend eine Lösung darstellen könnte, wurde im Rahmen des Projekts von verschiedenen Stakeholdern kritisch diskutiert und weiterführende Faktoren für die flächendeckende Umsetzbarkeit benannt (z. B. zeitnahe Einpflegung, Kompatibilität mit unterschiedlichen bestehenden Softwarelösungen).

Auch innerhalb der Zentren können Schnittstellen durch eine zentrale Stelle zur Koordination, durch SOPs und einen geregelten und zeiteffektiven Datentransfer günstig gestaltet werden, um so die Grundlage für eine gute Zusammenarbeit zwischen den Institutionen eines ZSE zu schaffen.

Insbesondere für Patient:innen mit unklaren Diagnosen, aber auch für diagnostizierte Patient:innen mit fehlender Spezialversorgung kann der Zugang zu einem ZSE wichtig sein. Für den Zugang stellen eine vorhandene Verdachtsdiagnose, die Bekanntheit der ZSE sowie klare, barrierefrei zugängliche Informationen Voraussetzungen dar. Wie bereits im Maßnahmenkatalog des NAMSE dargestellt, können technische Tools Primärversorgende in diesen Aspekten unterstützen [12]. Häufig werden Patient:innenorganisationen als wichtige Instanz beim Zugang zu Spezialist:innen genannt [1]. Es muss aber auch gewährleistet sein, dass Patient:innen, die nicht über eine Patient:innenorganisation informiert werden, zeitnah Informationen bzgl. der Spezialversorgung erhalten.

Während der Durchführung des Projekts ESE-Best und der einzelnen Erhebungsphasen wurden neben den Herausforderungen im Schnittstellenmanagement auch die Rahmenbedingungen, wie zum Beispiel die Finanzierung der Zentren und damit der Versorgung, thematisiert. In einer aktuellen Studie dazu wird geschlussfolgert, dass die bereits geschaffenen Vergütungsmöglichkeiten nicht ausreichen, um die „Komplexität und den überdurchschnittlichen Versorgungsaufwand Seltener Erkrankungen“ abzubilden [36]. In den Ergebnissen der Studie zeigt sich, dass die unzureichende Vergütungssituation nach Einschätzung von Zentren- und Patientenvertreter:innen die Versorgung negativ beeinflusst. Beispielsweise kann sich Frustration bei den Versorgenden entwickeln oder es fehlen Kapazitäten zum Aufbau von Versorgungsstrukturen. Es ist anzunehmen, dass sich solche Faktoren auch auf die Gestaltung der Schnittstellen auswirken, weil diese in der Regel v. a. von personellen und zeitlichen Ressourcen abhängig ist. Eine solche unzureichende Versorgungssituation kann dazu führen, dass Schnittstellenaufgaben an die Betroffenen übertragen werden. Es ist zu vermuten, dass so insbesondere für Personen mit geringerer Krankheitsmanagement- und Gesundheitskompetenz Nachteile in der Versorgung entstehen.

Bezüglich der abgeleiteten Empfehlungen müssen verschiedene Limitationen berücksichtigt werden. Im Rahmen der Bestandsaufnahme haben sich nur 24 von zu Projektbeginn identifizierten 32 ZSE beteiligt. Die Bestandsaufnahme erfolgte kurz nach dem Beschluss des Gemeinsamen Bundesausschusses (G-BA) zur Erstfassung der Regelungen der Aufgaben der ZSE [37] und noch vor dem Inkrafttreten der geänderten Regelungen im April 2022 [38]. Möglicherweise hat dies die Teilnahmebereitschaft beeinflusst. Bei der Gewinnung der Betroffenen für die Befragung nahmen nur 3 von 6 ausgewählten Best-Practice-Standorten teil. Die teilnehmenden Betroffenen waren dabei überwiegend einem mittleren und hohen sozioökonomischen Status zuzuordnen. Die Befragung der Primärversorgenden konnte aufgrund von unterschiedlichen Barrieren (u. a. Datenschutz) nicht wie geplant umgesetzt werden. Durch die Freiwilligkeit der Teilnahme an allen Studienphasen liegt ein Selektionseffekt vor. Gleichzeitig konnten durch den Einbezug der Perspektiven der ZSE, der Patient:innen bzw. Eltern sowie der Primärversorgenden bei der Entwicklung der Empfehlungen alle Bereiche der relevanten Schnittstellen adressiert werden. Darüber hinaus ermöglichten die abschließende Diskussion in einem Expert:innen-Workshop sowie die schriftliche Konsensrunde eine Einschätzung der Empfehlungen aus unterschiedlichen Perspektiven. Der Prozess der Entwicklung der Empfehlungen wurde in Anlehnung an ein Konsensverfahren zur Leitlinienentwicklung durchgeführt, folgte aber, wie auch im Projektplan vorgesehen, einem vereinfachten Vorgehen.

Das Projekt ESE-Best hat mittels einer Bestandsaufnahme der tatsächlich umgesetzten Versorgung von Menschen mit Seltenen Erkrankungen die Best-Practice-Ansätze identifiziert und auf Basis der erkannten Stärken und Schwächen des gelebten Schnittstellenmanagements Empfehlungen entwickelt. Es zeigt sich, dass sich einige Punkte des Beschlusses des G‑BA [37, 38] in den im Projekt entwickelten Empfehlungen wiederfinden. Dies betrifft beispielsweise strukturelle und personelle Anforderungen (z. B. Anlaufstelle mit fester Sprechzeit). Die darüber hinaus formulierten Empfehlungen fokussieren sich auf die Ausgestaltung der Strukturen, aber auch auf übergeordnete Aspekte, die für ein gelungenes Schnittstellenmanagement essenziell sind. Die Empfehlungen können ZSE und relevante Stakeholder unterstützen, die Umsetzung von Schnittstellen zu planen und die dabei wichtigen Aspekte im Blick zu haben. Auf diese Weise kann eine adäquate, patient:innenzentrierte Versorgung erfolgen. Ein Teil der Empfehlungen setzt voraus, dass Primärversorgende vorhandene Informationsstrukturen (z. B. SE-Atlas, Orphanet) und Organisationsstrukturen (z. B. Zentrenstruktur) kennen. Eine aktuelle Studie zeigt, dass dies nicht vorausgesetzt werden kann [39]. Vor diesem Hintergrund sind Konzepte zur Bekanntmachung der Strukturen und zur Sensibilisierung der Primärversorgenden für Seltene Erkrankungen eine wichtige Ergänzung.

## Fazit

Die Versorgung von Menschen mit Seltenen Erkrankungen erfordert neben medizinischer Expertise ein gut funktionierendes Schnittstellenmanagement, um Patient:innen, aber auch Primärversorgende und ZSE beim Erkrankungsmanagement sowie bei der Informationsweitergabe zu entlasten. Als wichtige Aspekte von Schnittstellen im Kontext Seltener Erkrankungen wurden die Zusammenarbeit zwischen ZSE und Primärversorgung, Schnittstellen innerhalb der ZSE und die Schnittstelle ZSE und Patient:innen, aber auch die Steigerung der Bekanntheit von SE und weiterführende Aspekte (z. B. Schaffung von Koordinierungs- und Informationsstrukturen) identifiziert. Im Rahmen des Projekts ESE-Best wurden hierzu Empfehlungen abgeleitet, die die Umsetzung einer patient:innenzentrierten Versorgung unterstützen. Es ist dabei zu bedenken, dass zeitliche und personelle Ressourcen sowie organisationale Strukturen die Schnittstellenarbeit im Einzelfall beeinflussen können. Die Umsetzungsempfehlungen können an örtliche Gegebenheiten adaptiert werden.

## References

[1] Hoffmann GF, Mundlos C, Dötsch J (2020). Seltene Erkrankungen in der Pädiatrie – von der Diagnostik und Behandlung einzelner Erkrankungen zum Aufbau von Netzwerkstrukturen. Monatsschr Kinderheilkd.

[2] Neff M, Schaaf J, Tegtbauer N (2021). se-atlas.de – Versorgungsatlas für Menschen mit Seltenen Erkrankungen. Internist.

[3] de la Paz MP, Taruscio D, Groft SC (2017). Rare diseases epidemiology: update and overview.

[4] Nguengang Wakap S, Lambert DM, Olry A (2019). Estimating cumulative point prevalence of rare diseases: analysis of the orphanet database. Eur J Hum Genet.

[5] Häberle J, Burlina A, Chakrapani A (2019). Suggested guidelines for the diagnosis and management of urea cycle disorders: first revision. J Inherit Metab Dis.

[6] Boy N, Mühlhausen C, Maier EM (2017). Proposed recommendations for diagnosing and managing individuals with glutaric aciduria type I: second revision. J Inherit Metab Dis.

[7] Mueller T, Jerrentrup A, Bauer M, Fritsch H, Schaefer J (2016). Characteristics of patients contacting a center for undiagnosed and rare diseases. Orphanet J Rare Dis.

[8] Llubes-Arrià L, Sanromà-Ortíz M, Torné-Ruiz A, Carillo-Álvarez E, García-Expósito J, Roca J (2022). Emotional experience of the diagnostic process of a rare disease and the perception of support systems: A scoping review. J Clin Nurs.

[9] Tumiene B, Graessner H (2021). Rare disease care pathways in the EU: from odysseys and labyrinths towards highways. J Community Genet.

[10] Stoller J (2018). The challenge of rare diseases. Chest.

[11] Pauer F, Pflaum U, Lührs V, Frank M, von der Schulenburg J (2016). Die Versorgung von Menschen mit seltenen Erkrankungen in Niedersachsen: Ergebnisse einer Ärztebefragung. Z Evid Fortbild Qual Gesundhwes.

[12] Nationales Aktionsbündnis für Menschen mit Seltenen Erkrankungen (2013) Nationaler Aktionsplan für Menschen mit Seltenen Erkrankungen. https://www.bundesgesundheitsministerium.de/fileadmin/Dateien/3_Downloads/N/NAMSE/Nationaler_Aktionsplan_fuer_Menschen_mit_Seltenen_Erkrankungen_-_Handlungsfelder__Empfehlungen_und_Massnahmenvorschlaege.pdf. Zugegriffen: 9. Sept. 2022

[13] Sakaguchi F, Lenert L (2015). Improving continuity of care via the discharge summary. AMIA Annu Symp Proc.

[14] Kripalani S, LeFevre F, Phillips CO, Williams MV, Basaviah P, Baker DW (2007). Deficits in communication and information transfer between hospital-based and primary care physicians: implications for patient safety and continuity of care. JAMA.

[15] Ommen O, Ullrich B, Janßen C, Pfaff H (2007). Die ambulant-stationäre Schnittstelle in der medizinischen Versorgung. Probleme, Erklärungsmodell und Lösungsansätze. Med Klin.

[16] Bundesministerium für Gesundheit Richtlinie Qualitätsmanagement – Richtlinie des Gemeinsamen Bundesausschusses über grundsätzliche Anforderungen an ein einrichtungsinternes Qualitätsmanagement. https://www.g-ba.de/downloads/62-492-2309/QM-RL_2020-09-17_iK-2020-12-09.pdf. Zugegriffen: 9. Sept. 2022 (für Vertragsärztinnen und Vertragsärzte, Vertragspsychotherapeutinnen und Vertragspsychotherapeuten, medizinische Versorgungszentren, Vertragszahnärztinnen und Vertragszahnärzte sowie zugelassene Krankenhäuser BAnz AT 15.11.2016 B2)

[17] Schulz H, Barghaan D, Harfst T, Dirmaier J, Watzke B, Koch U (2006). Versorgungsforschung in der psychosozialen Medizin. Bundesgesundheitsblatt Gesundheitsforschung Gesundheitsschutz.

[18] Osterloh F (2013). Nur durch ständigen Dialog hat sich manches verändert: Mit einem guten Entlassmanagement können Schneisen durch den Versorgungsdschungel geschlagen werden – zum Vorteil von Patienten und Leistungserbringern. Drei Beispiele aus der Praxis. Dtsch Ärztebl.

[19] Fleck K (2012). Stationär-ambulante Schnittstellen: Wie Entlassungsmanagement hilft, Versorgungslücken zu vermeiden. Berl Arzte.

[20] Stieber C, Mücke M, Windheuser I (2017). Kurze Wege zur Diagnose. Eine Handlungsempfehlung für Patienten ohne Diagnose. Bundesgesundheitsblatt Gesundheitsforschung Gesundheitsschutz.

[21] Mehrmann L, Schwarz S (2013). Nahtloser Übergang. Checklisten für das ärztliche Schnittstellenmanagement zwischen den Versorgungssektoren werden aktualisiert (Interview). Qualitas.

[22] Zigmond AS, Snaith RP (1983). The hospital anxiety and depression scale. Acta Psychiatr Scand.

[23] Whoqol Group (1998). Development of the World Health Organization WHOQOL-BREF quality of life assessment. Psychol Med.

[24] Lehmann C, Koch U, Mehnert A (2012). Psychometric properties of the German version of the short-form supportive care needs survey questionnaire (SCNS-SF34-G). Support Care Cancer.

[25] Pelentsov LJ, Fielder AL, Laws TA, Esterman AJ (2016). Development of the parental needs scale for rare diseases: a tool for measuring the supportive care needs of parents caring for a child with a rare disease. J Multidiscip Healthc.

[26] Ahgren B, Axelsson S, Axelsson R (2009). Evaluating intersectoral collaboration: a model for assessment by service users. Int J Integr Care.

[27] Scholl I, Hölzel L, Härter M, Dierks M, Bitzer E, Kriston L (2011). Fragebogen zur Zufriedenheit in der ambulanten Versorgung – Schwerpunkt Patientenbeteiligung (ZAPA). Klin Diagn Eval.

[28] Schmidt J, Lamprecht F, Wittmann W (1989). Zufriedenheit mit der stationären Versorgung. Entwicklung eines Fragebogens und erste Validitätsuntersuchungen. Psychother Med Psychol.

[29] Bédard M, Molloy D, Squire L, Dubois S, Lever J, O’Donnell M (2001). The Zarit Burden Interview: a new short version and screening version. Gerontologist.

[30] Brouwer W, Van Exel N, Van Gorp B, Redekop W (2006). The CarerQol instrument: a new instrument to measure care-related quality of life of informal caregivers for use in economic evaluations. Qual Life Res.

[31] Varni J, Seid M, Rode C (1999). The PedsQL™: measurement model for the pediatric quality of life inventory. Med Care.

[32] Nationales Aktionsbündnis für Menschen mit Seltenen Erkrankungen (2019). Statusbericht zur Umsetzung des Nationalen Aktionsplans für Menschen mit Seltenen Erkrankungen.

[33] Rillig F, Grüters A, Schramm C, Krude H (2022) The interdisciplinary diagnosis of rare diseases: results of the Translate-NAMSE project. Dtsch Ärztebl Int 119:469–475. 10.3238/arztebl.m2022.0219 10.3238/arztebl.m2022.0219 PMC9664985 35635437

[34] Choukair D, Hauck F, Bettendorf M (2021). An Integrated clinical pathway for diagnosis, treatment and care of rare diseases: model, operating procedures, and results of the project TRANSLATE-NAMSE funded by the German Federal Joint Committee. Orphanet J Rare Dis.

[35] Mosquera R, Avritscher E, Samuels C (2014). Effect of an enhanced medical home on serious illness and cost of care among high-risk children with chronic illness: a randomized clinical trial. JAMA.

[36] Litzkendorf S, Eidt-Koch D, Zeidler J (2022). Nachhaltige Vergütung der B-Zentren für Seltene Erkrankungen in Deutschland – Status quo und Lösungsansätze. Bundesgesundheitsblatt Gesundheitsforschung Gesundheitsschutz.

[37] Gemeinsamer Bundesausschuss Regelungen zur Konkretisierung der besonderen Aufgaben von Zentren und Schwerpunkten gemäß § 136c Absatz 5 SGB V (Zentrums-Regelungen) BAnz AT 12.03.2020 B2. https://www.g-ba.de/beschluesse/4072/. Zugegriffen: 9. Sept. 2022

[38] Gemeinsamer Bundesausschuss Regelungen zur Konkretisierung der besonderen Aufgaben von Zentren und Schwerpunkten gemäß § 136c Absatz 5 SGB V BAnz AT 03.06.2022 B3. https://www.g-ba.de/richtlinien/117/. Zugegriffen: 9. Sept. 2022

[39] Druschke D, Krause F, Müller G, Scharfe J, Hoffmann GF, Schmitt J (2021). Potentials and current shortcomings in the cooperation be tween German centers for rare diseases and primary care physicians: results from the project TRANS- LATE-NAMSE. Orphanet J Rare Dis.

